# Estimates of Genetic Parameters for Shape Space Data in Franches-Montagnes Horses

**DOI:** 10.3390/ani12172186

**Published:** 2022-08-25

**Authors:** Annik Imogen Gmel, Alexander Burren, Markus Neuditschko

**Affiliations:** 1 Animal GenoPhenomics, Agroscope, Rte de la Tioleyre 4, 1725 Posieux, Switzerland; 2 Equine Department, Vetsuisse Faculty, University of Zurich, Winterthurerstrasse 260, 8057 Zurich, Switzerland; 3 School of Agricultural, Forest and Food Sciences HAFL, Bern University of Applied Sciences, Länggasse 85, 3052 Zollikofen, Switzerland

**Keywords:** horse, breeding, heritability, conformation, joint angles, geometric morphometrics

## Abstract

**Simple Summary:**

Equine breeding is often based on conformation traits, describing the proportions, shape and joint angles of a horse. These conformations traits are, however, mostly subjectively judged and not measured objectively, affecting the response of selection through lower heritabilities and precision. In this study, we measured joint angles, quantified the variation in shape of 608 Swiss Franches-Montagnes (FM) horses and estimated the heritability of these traits. We found that the poll angle had the highest heritability of all joint angles (h^2^ = 0.37), and variation in shape describing the type (heavy–light) was also fairly heritable (h^2^ = 0.36–0.37). Furthermore, the shape of the FM stallions has clearly evolved towards a lighter type from 1940 to 2018 without stabilisation in recent years, risking the loss of the light draught horse type. Phenotyping based on photographs allowed us to improve the accuracy of certain joint angle traits, and to monitor the conformational development of the FM breed.

**Abstract:**

Conformation traits such as joint angles are important selection criteria in equine breeding, but mainly consist of subjective evaluation scores given by breeding judges, showing limited variation. The horse shape space model extracts shape data from 246 landmarks (LM) and objective joint angle measurements from triplets of LM on standardized horse photographs. The heritability was estimated for 10 joint angles (seven were measured twice with different LM placements), and relative warp components of the whole shape, in 608 Franches-Montagnes (FM) horses (480 stallions, 68 mares and 60 geldings born 1940–2018, 3–25 years old). The pedigree data comprised 6986 horses. Genetic variances and covariances were estimated by restricted maximum likelihood model (REML), including the fixed effects birth year, age (linear and quadratic), height at withers (linear and quadratic), as well as postural effects (head, neck, limb position and body alignment), together with a random additive genetic animal component and the residual effect. Estimated heritability varied from 0.08 (stifle joint) to 0.37 (poll). For the shape, the type was most heritable (0.36 to 0.37) and evolved from heavy to light over time. Image-based phenotyping can improve the selection of horses for conformation traits with moderate heritability (e.g., poll, shoulder and fetlock).

## 1. Introduction

Conformation traits are important in equine breeding as they have been associated with health, longevity and performance [1,2,3,4,5,6,7,8]. Conformation is a phenome and encompasses relatively objective traits such as the length of a body segment or an angle between joints, but also highly subjective traits such as the type (breed type, sex type) and the shape of the head, withers, back or croup. Many horse breeding associations perform conformation evaluations at breeding shows by subjective assessment by breeding experts on scoring sheets (e.g., subjective valuating scores, linear profiling) and/or through measurements made on the living animal (e.g., withers height, croup height, limb length). These data commonly provide the basis for the analysis of variance components and subsequent breeding value estimation in European sport horse breeds [9,10]. 

Despite their widespread use, essentially due to their simple implementation, conformation traits based on subjective assessments, including linear profiling, exhibited certain limitations. In many studies, the scale of the scoring scheme was not used in full, leading to poorly distributed data tending towards the optimum, while the lower extreme of the scale was avoided [11,12,13]. Furthermore, the inter-rater reliability between experts on the breed (judges) who routinely assessed conformation traits in horses was highly variable depending on the breed: the repeated assessments of linear profiling traits in the Pura Raza Español were highly correlated based on intra-class correlation coefficients (ICCs; 0.96 < ICC < 0.99) [14], indicating reliable data. However, the repeatability of conformation traits that were judged on 4306 Finnhorse and 294 Standardbred trotter foals was estimated using a correlation coefficient (r) that only ranged from 0.06 to 0.48 in Standardbred trotters and from 0.24 to 0.38 in Finnhorses [15], respectively. Furthermore, multiple experts simultaneously assessing the same Lipizzaner horses showed poor inter-rater reliabilities based on the kappa statistic (0.06 < κ < 0.49) [16,17]. The reliability of expert scores is seldom reported as many horse breed associations only assess the conformation of horses once in their lifetimes. Hence, specific reliability studies are rare, making it difficult to assess the quality of the scoring data. 

The Franches-Montagnes (FM) horse is the last native Swiss breed. In its history that spans over one hundred years, the breed has evolved from a draught horse used for tilling fields to the light draught horse breed used for leisure known today [18]. To be able to perform these different functions, the conformation of the FM had to adapt over time, and conformation traits were used empirically to move towards modern breeding goals. Since 1990, three-year-old FM horses have been presented in hand and scored for 19 conformation and five locomotor traits on a linear profiling scale by experts of the breed. However, the distribution of the scores does not always follow a normal distribution, with the lowest scores being avoided, and the mean tending towards the perceived optimum (9) instead of the scale median (5) [19]. The same trend could be observed for gait quality traits [20,21]. Furthermore, FM experts scoring the same horses simultaneously, either in hand or on the treadmill over video recordings, had poor agreement (ICC < 0.50) for all scored gait quality traits [20,21]. The heritability for conformation traits in the FM ranged from 0.09 (length of shoulder, hind limb muscle) to 0.79 (height at withers, measured) [22]. 

The heritability of conformation traits naturally depends on the breed, the sample size used for the calculation, the method of data collection (subjective evaluation, linear profiling score or measurement), and the underlying genetic architecture. However, some tendencies are similar between breeds. Based on a meta-analysis of 30 studies on genetic parameter estimates of conformation traits in horses, height at croup and height at withers had the highest heritability for measured conformation traits (h^2^ = 0.61 for height at croup and h^2^ = 0.58 for height at withers) and were highly genotypically correlated (r_g_ = 0.94) [23]. The size of the horse is routinely measured, with data available from many horses and breeds, and the genetic architecture has also been shown to be highly responsive to selection as most of the variation in the height at withers is essentially determined by four genes [24]. Furthermore, objective measures are generally more heritable compared to corresponding scored traits [23]. This may be at least partially due to lower subjectivity in the data collection. However, one key source of error in measuring conformation traits is the landmark definition, i.e., which anatomical structures should be used as reference points, and whether they can be easily identified [25].

The horse shape space model proposed by Druml et al. [16] uses standardized photographs to extract shape data from landmarks on fixed anatomical structures, and semi-landmarks equidistantly placed on curves so that they can then be analysed as landmarks. This method was first developed in the Lipizzaner [16,17] and then applied to the FM horse [26]. While only the overall shape was initially analysed, joint angle measurements were later extracted from the initial landmarks. When comparing linear profiling traits describing, e.g., the shoulder or croup incline with objectively measured joint angles from the horse shape space model, the two corresponding traits were not significantly associated, suggesting that the expert scores do not represent the variation that is objectively quantified using the horse shape space model [26]. In the initial horse shape space model [16], the majority of landmarks used to extract joint angle measurements were placed on the surface of the horse (i.e., in front of the joint), as that made them easier to place. However, placing the landmarks in the centre of the joint is expected to improve the predictive relationship between conformation and joint movement, provided they can be placed as accurately as those placed in front of the joints can. 

The aims of the study were to estimate variance components and heritability for conformation traits extracted from the horse shape space model in the FM horse breed. Using different landmark settings, joint angles were evaluated both for their heritability and repeatability. Finally, the evolution of the conformation of the FM breed was visualized for the period from 1940 to 2018. 

## 2. Materials and Methods

### 2.1. Phenotypes

**Figure 1:**
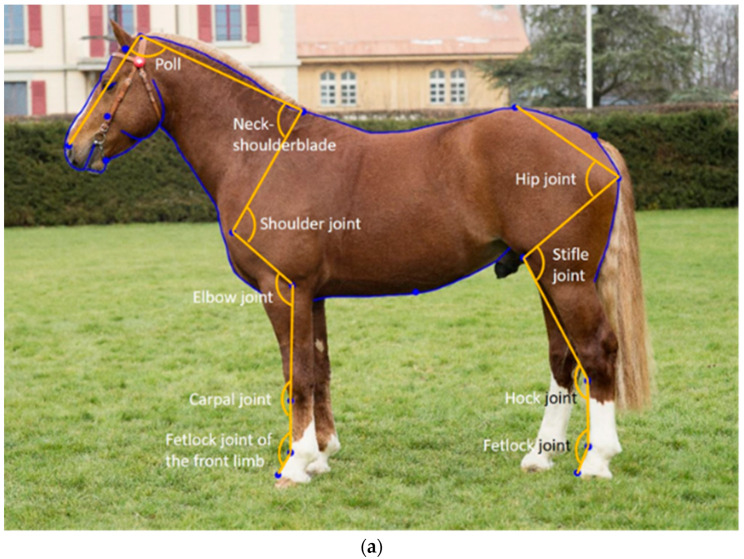

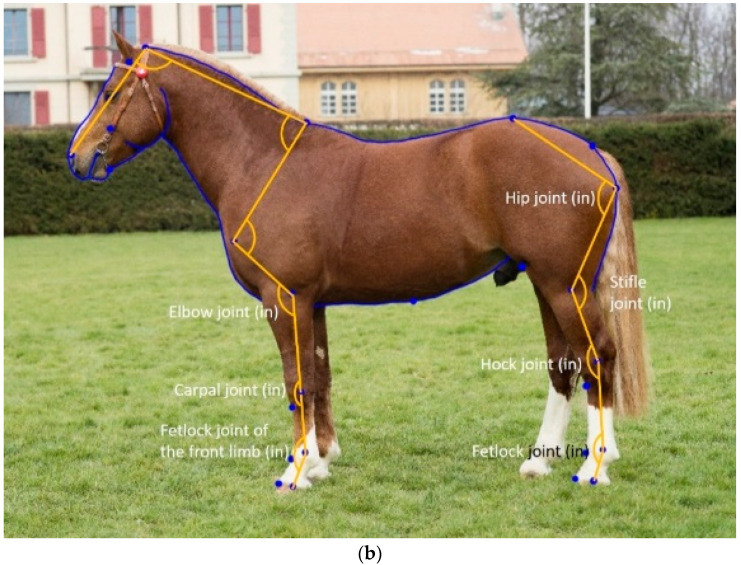
Example of the initial horse shape space model with both curves and angles derived from landmarks (**a**), and the newly proposed landmarks and additional joint angles (**b**).

One photograph for each of the 608 FM horses was selected from the archives of the Swiss National Stud Farm or collected on farms under the permits VD3096 and VD3527b. The horses were born between 1940 and 2018 (median = 2005) and aged between 3 and 25 years on the photographs (median = 3). Age was unknown for 133 horses. In total, 480 stallions, 68 mares and 60 geldings were included in the analysis. The horses were positioned in an open posture for the photograph (Figure 1). To account for individual variation, the posture of the horses on the photographs was classified based on previously described criteria [26]: head height, head position in relation to the camera, front limb position, hind limb position and body alignment to the camera.

Conformational data were extracted from the photographs based on the horse shape space model published by Druml et al. [16], consisting of both curves and landmarks, using the digitising tool tpsDig2 version 1.78 [27]. The semi-landmarks on the curves were placed at equal distances within each curve, with the final horse model consisting of 246 landmarks (Figure S1). All photographs were digitised by the same person. The raw landmark coordinates were first normalised using a Generalised Procrustes Analysis (GPA). We then used a principal component analysis (PCA) of the normalised landmarks to convert the shape data into relative warp scores (PCs; the principal components of the partial warp matrix), explaining the main variation in the data. We considered only the first five PCs for the analysis of variance components, which were visualised in warp grids using tpsRelw version 1.70 [28]. 

From the proposed 246 landmarks, 10 joint angle measurements can be extracted: namely the poll angle, neck–shoulder blade angle, shoulder joint angle, elbow joint angle, carpal angle, the fetlock joint angle of the front limb, hip joint angle, stifle joint angle, hock joint angle, and fetlock joint angle of the hind limb (Figure 1a). The landmarks for measuring the limb angles (from elbow to fetlock in the front limb and from hip to fetlock in the hind limb) were placed in front of the joint [26]. As an extension to the initial model, we proposed seven additional landmarks, to measure the same angles of the limbs, but with the landmarks located in the centre of the joints when looking from the side (Figure 1b, Table 1). All angles were calculated in R [29] using a custom-made script. Mean differences in joint angle measurements due to landmark placements were analysed using a paired *t*-test. To evaluate the repeatability of landmark placements for the old versus the new proposed joint angle measurements, the photographs of 480 horses were digitised in triplicate by the same digitiser. The repeatability of the joint angle measurements was estimated with an intra-class correlation coefficient (ICC). For these 480 horses, the mean landmarks were calculated before performing the GPA with the rest of the horses digitised only once. 

The effects of posture, age, sex and year of birth on the measurements were evaluated using a linear model; each joint angle and PC as outcome variables; and all posture variables (head height, head position in relation to the camera, front limb position, hind limb position and body alignment), age, sex (mare, gelding or stallion) and year of birth, as fixed effects.

### 2.2. Evolution of Conformation Traits over Time

Finally, the evolution over time of the different measurements in stallions (to avoid the sex effect) was evaluated. A second set of linear models was computed for the stallion subsample with the effects of posture, age and year of birth as fixed effects (excluding sex). Each of the joint angles and PCs was plotted against the year of birth over time, with a trend line computed in ggplot2 as a loess function (local polynomial regression fitting). Pairs of joint angles were plotted in the same graph for comparison. 

### 2.3. Animal Model

The following multivariate individual animal model was applied to estimate variance components (VC) for 17 joint angles. The same model was used to estimate VC for the five principal components:
y_ijklmnopqr_ = YOB_i_ + Age_j_ + WH_k_ + Head_camera_l_ + Head_height_m_ + Front_limb_n_ + Hind_limb_o_ + Body_p_ + a_q_ + e_ijklmnopq_


with:
y_ijklmnopq_consecutive observation on a trait

fixed effects:
YOB_i_year of birthAge_j_age of horse at collection in years (linear and quadratic)WH_k_height at withers (linear and quadratic)Head_camera_l_head position in relation to the cameraHead_height_m_head heightFront_limb_n_front limb positionHind_limb_o_hind limb positionBody_p_body alignment to the camera

random effects:a_q_horse qe_ijklmnopq_random residual

Phenotypic and genetic variance components were estimated by REML using the software ASReml 4.2 [30]. For each trait, heritability (h^2^) was calculated as follows:

$$h^{2}=\sigma_{a}^{2}/\left(\sigma_{a}^{2}+\sigma_{e}^{2}\right)$$

where $\sigma_{a}^{2}$ is the additive genetic variance and $\sigma_{e}^{2}$ is the residual variance. 

For the PCs, the ASReml US variance structure was used for the assessment of genetic and residual variance. The use of the US variance structure for the joint angle traits did not result in positive definite variance structure, which is why the model did not converge. Therefore, an ASReml XFA4 variance structure was used for the additive genetic variance.

In total, the pedigree file contained 6986 horses, with 1663 sires (219 founders) and 4991 dams (436 founders). The mean pedigree completeness, considering 1 to 5 generations, was 71.59%.

## 3. Results

### 3.1. Descriptive Statistics and Comparisons between Joint Angle Measurements

The two types of fetlock joint angles of the hind limb exhibited the broadest range (42.50–43.30°, difference between maxima and minima), while both types of carpal joint angles had the smallest range of the joint angles (14.40–15.50°, Table 2). The highest repeatability was for the poll angle (ICC = 0.99), while the lowest was for the fetlock joint of the front limb (ICC = 0.66). For the pairs of joint angles (elbow joint, carpal joint, fetlock joint of the front limb, hip joint, stifle joint, hock joint, and fetlock joint of the hind limb), the joint angles measured with the new landmarks within the joints were significantly larger (less acute, *p* < 0.0001) than the initial measurements based on a paired *t*-test.

### 3.2. Postural Effects on the Joint Angles

Each joint angle was significantly affected by at least one postural variable (Table 3). Pairs of joint angle measurements (differing only in the landmark placement) were not affected by the same combination of postural variables, except the two types of elbow and hip joint angles.

### 3.3. Visualisation of the Principal Components of Shape Variation

**Figure 2:**
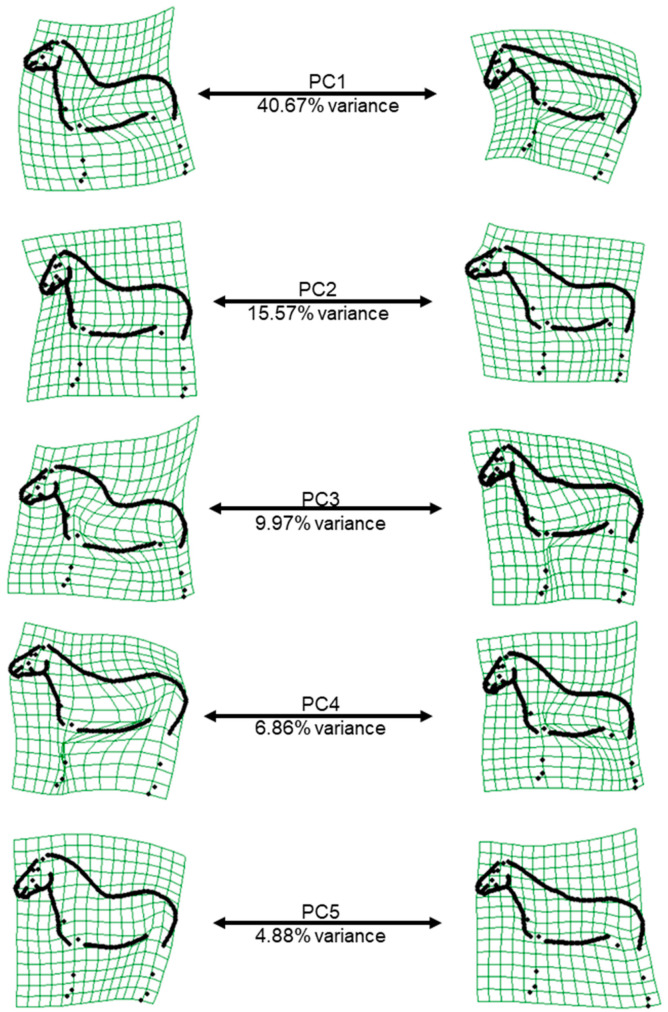
Representation of the extreme shapes describing the first five relative warp axes on warp grids from low (**left**) to high (**right**), with the percentage of explained variance under the arrow.

Based on the visualisation of the minima and maxima of the PCs, we can infer that PC1 represents the shape variation due to head height, and PC2 the variation due to the angle at the poll (flexion-extension) (Figure 2). PC3, PC4 and PC5 essentially represent the musculature of the neck. In addition, PC3 also represents the shape of the withers and back, PC4 and PC5 the shape of the croup and the position of the front and hind limbs.

### 3.4. Postural Effects on the Principal Components of Shape Variation

In the shape variation (PC1 to PC5), there were no significant differences between mares and geldings, but highly significant differences between mares and stallions (Table 4). Head height significantly affected the first four PCs, while the position of the front limb, hind limb and body alignment had a significant effect on all PCs presented here. PC1, the principal component explaining most of the variance in this dataset (41%), was significantly affected by all posture variables.

### 3.5. Evolution of the Breed

**Figure 3:**
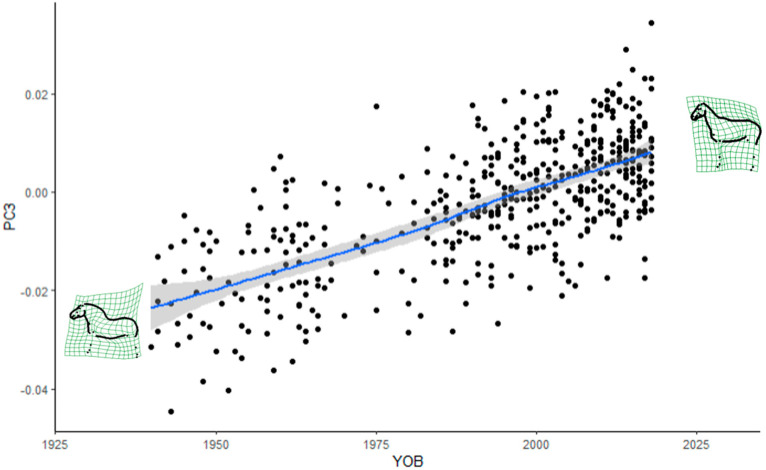
Evolution of the third relative warp score (PC3) in stallions born from 1940 to 2018 (YOB), with the extreme shapes on relative warp grids, with the trend line (in blue, with the confidence interval in light grey) from local polynomial regression fitting.

We recalculated the linear regression models for postural and other variables with only stallions (File S1). Based on the linear regression models, year of birth most significantly affected PC3, which increased linearly over time, meaning that the stallions are evolving towards a lighter type (Figure 3). Year of birth also significantly affected PC1, showing a sigmoid curve, and PC5, slightly increasing from the 2000s (Figure S2). 

Year of birth had a significant effect on all joint angles except the carpal angle, the fetlock joint of the hind limb and the stifle joint (both measured with the landmarks inside the joint) as well as the hock joint (measured in front of the joint, Figures S3–S5). The poll angle decreased until the mid-1990s and then increased again slightly up to 2018. The neck–shoulder blade angle increased almost linearly over time. The shoulder joint angle increased slightly up to the mid-2000s, when it reached a plateau. Both types of elbow joint angles followed a parallel trend line, increasing from 1940 to 1960 and decreasing until 2000 to reach a plateau. The carpal joint angle (measured in front of the joint) decreased until 1980, increased until 2000, and has been decreasing again until 2018. The fetlock joint of the front limb (measured in front of the joints) and both types of hip joint measurements remained stable until the 2000s, with a decrease in recent years. The stifle joint (measured in front of the joints) increased slightly until the 1980s, decreased until the mid-2000s and is currently on a slight uphill tendency. The hock joint (measured in front) appeared to decrease around 1990 and stabilise at a lower level. The fetlock joint of the hind limb (measured in front) appeared to remain stable over time, with a slight decrease after 2000 and up to recent years. The individual plots of all measurements in stallions against the year of birth were summarised in Figures S2–S5.

### 3.6. Genetic Analyses of Joint Angle Measurements

For the joint angles, the highest heritability was found for the poll angle (h^2^ = 0.37 ± 0.09) and the lowest for the hock joint (with landmarks within the joints, h^2^ = 0.08 ± 0.05, Table 5). Most genetic, phenotypic and residual variances are >1, except the genetic additive variance for both types of carpal joint, the stifle joint (with landmarks in front of the joint) and both types of hock joint. 

The highest genotypic and phenotypic correlations were between the two elbow angles, representing virtually the same trait (r_p_ = 0.99, r_g_ = 0.99, Table 6). Relatively high (0.70–0.90) phenotypic correlations were also present between the same joint angles measured differently (hip, stifle, hock and fetlock joint of the hind limb), except between the two carpal joint angles and the two front limb fetlock joint angles (0.45 and 0.69, respectively). The highest phenotypic correlation between two different joints was between the shoulder and elbow joints (measured from the centre of the limb, r_p_ = 0.64). In absolute values, the lowest genotypic correlation was between the hip and hock joint angles measured in front of the joint (r_g_ = 0.01). Apart from the near perfect correlation between the two elbow angles, the highest genotypic correlation was between the hip and stifle joint angles (r_g_ = 0.96).

### 3.7. Genetic Analyses of the Principal Components of Shape Variation

For the relative warp components, the variance components were very small (Table 7). PC5 had the highest heritability (h^2^ = 0.37 ± 0.09), and PC1 the lowest (h^2^ = 0.13 ± 0.08).

The highest phenotypic correlation was between PC1 and PC3 (r_p_ = 0.41, Table 8). The fourth and fifth PCs showed low phenotypic correlations to the other PCs. The highest genetic correlation was between PC1 and PC2 (r_g_ = −0.55). The genotypic correlations ranged from 0.06 to 0.55 in absolute values. 

## 4. Discussion

The joint angles with the highest heritability (h^2^) were the poll and the fetlock joint of the front limb (Table 5). The high h^2^ of the poll angle was consistent with results from a multi-breed genome-wide association study of the same joint angles from 300 FM and 224 Lipizzaner horse photographs [31]. The best-associated quantitative trait locus (QTL) was the poll angle on equine chromosome (ECA) 28, near the gene *ALX1*, associated with cranial morphology [32]. The genome-wide h^2^ for the poll angle in the two breeds was h^2^ = 0.38, nearly equal to the pedigree-based h^2^ estimated here. In contrast, the highest genome-wide h^2^ for Lipizzaner and FM was for the fetlock joint of the hind limb (h^2^ = 0.58) and a suggestive QTL on ECA 27, whereas this trait had a much lower pedigree-based h^2^ = 0.19 in the FM.

The placement of the landmarks for the calculation of the joint angles changed the acuteness of the joint angles. For all pairs of joint angles, the landmark placement inside the joint was significantly larger (less acute) than when the landmarks were placed in front of the joint (old model). However, the phenotypic correlation is more relevant to understand whether the landmark placement affects the anatomical meaning of the measurement. For example, the elbow joint remained virtually identical whether the third landmark was placed in front or within the carpal joint, as shown by near-perfect genetic and phenotypic correlations between the two elbow joint measurements (Table 6), and a nearly identical repeatability (ICC, Table 2) and h^2^ (Table 5). The two types of elbow joint angles were also significantly affected by the same combination of posture and other external variables (age, sex and year of birth, Table 3). For this angle, it makes no difference which landmark placement to use. The two types of carpal joint angles were the least phenotypically correlated of the paired joint angles (Table 6), the angle from the old model (landmark in front of the joint) was significantly different between mares and stallions, while with the new landmark placement inside the joint, the sex difference was between mares and geldings (Table 4). The heritability for the new landmark placement was higher (Table 5), with a nearly identical ICC (Table 2). In this case, it makes more sense to measure the carpal joint with the new landmark placement. For the fetlock angle of the front limb, h^2^ (Table 5) and ICC (Table 2) were higher with the new landmark placement, affected by the age of the horse and not by the birth year (Table 4), as was the case for fetlock angle measured with the old landmark placement. For the joint angles of the front limb (elbow, carpus and fetlock), the new landmark placement increases h^2^ (Table 5) and ICC (or at least does not negatively affect the latter; Table 2).

The trends were less clear in the hind limbs. For the stifle and hock, ICC (Table 2) and h^2^ (Table 5) concurred, so that the angle that was measured the most accurately was also the one with the highest h^2^ (stifle with landmarks within the joints, hock with landmarks outside the joints). The results were more difficult to interpret for the hip and fetlock joint of the hind limb. For the hip joint, the ICC was only slightly higher, but the h^2^ lower, when using the centre of the stifle joint as the third landmark. However, the hip joint angle measured with the landmarks in front of the patella was additionally affected by the head height and the sex (mares were significantly different from geldings; Table 4) which suggests that the angle is more affected by the posture (i.e., environmental effects). While the ICC was lower for the fetlock joint of the hind limb (Table 2), the h^2^ was higher when using the landmarks in front of the joints (Table 5). Deciding which measurement is better for a particular joint is complicated in the case when h^2^ and ICC are not in lockstep. Another way to choose the most informative landmark placement might be to compare the joint angles with kinematic parameters, to assess whether one set of landmarks is a better predictor for movements associated with gait quality traits such as hind limb protraction [33], i.e., whether a certain joint angle measurement is more functionally relevant. Considering the low amount of additional effort involved in placing the supplementary landmarks, we currently recommend assessing the ICC and h^2^ of both types of joint angle measurements in other breeds using the two landmark settings, in order to optimise the results.

The first and second relative warp scores (PC1 and PC2) represented the shape variation induced by the head-neck position (PC1: head height, PC2: flexion-extension at the poll, Figure 2), which is why the posture variable head height was so strongly associated with PC1. For PC2 (as well as the poll angle), when the horse has its head turned towards the camera, the angle decreases, which explains the significant association between the variable “head in relation to camera” with PC2 and poll angle. In practice, the variation due to the head and neck position is hard to avoid, and this result was consistent with several previous horse shape space studies on FM and Lipizzaner horses [16,17,26]. While PC1 and PC2 explain most of the shape variation, they are less heritable than the three following PCs, reflecting the fact that the variation in shape mainly originates from the posture, which is an “environmental” effect. The moderate h^2^ of PC3, PC4 and PC5 is consistent with previous findings that the body type (heavy–light), quantified by bone thickness up to now, is the second highest source of conformational variation in the horse after height [34]. However, these PCs are also affected by posture, especially by the position of the front and hind limbs. Therefore, posture variables should always be considered when working with conformational data, although considering all the posture variables as fixed effects in the model of analysis might have caused an over-parameterization of the model, thereby affecting the accuracy of the estimates. The majority of additive genetic, phenotypic and residual variances were large (>1; Table 5), in contrast to other studies that included several thousand horses to estimate genetic parameters [12,13,14,15,23]. Furthermore, the standard errors for the genetic correlations, in particular, were large as well (Table 6 and Table 8). Reducing the postural variance in the data when photographing the horses and increasing the sample size should improve the accuracy of the estimates on the long term.

The current accuracy of data extracted from the horse shape space model still shows potential for selection on conformation traits based on this method, especially if a horse breeding association routinely records these traits. Of the 19 linear conformation traits routinely assessed by breeding experts in the FM, five describe joint angles we quantified here. We can therefore compare our h^2^ estimated for measured joint angles on 608 horses against the h^2^ estimates for 18,297 horses tested between 1994 and 2013 [22]. The scored trait “shoulder incline” (straight–inclined) had h^2^ = 0.09 [22], while the measured shoulder joint angle’s h^2^ was twice as high (Table 5). The trait “front limb” (back-at-the-knee–over-at-the-knee) had h^2^ = 0.14 [22], compared to the measured carpal joint angles (h^2^ = 0.13 measured in front of the joint, h^2^ = 0.27 within the joint), suggesting that this trait has the potential for improvement when using the measurement within the joint. As the range of the measurement is limited for both carpal joint angles, any slight error in landmark placement has a disproportionate effect on the accuracy of the measurement, as is shown by the lower ICC compared to, e.g., the hip joint angles (Table 2). The scored “croup incline” (horizontal–sloping) had h^2^ in the same range as the hip joint angles (h^2^ = 0.20 [22]). The measured hock joint angles exhibited a lower h^2^ than the “hock angle” trait (straight–angulated, h^2^ = 0.19, [22]). These traits were also not associated with each other in a previous direct comparison between measurements and scores [26]. In this case, the linear profiling score may be considered more useful for selection than the hock joint measurement. The final linear profiling trait with an equivalent joint angle is the “fetlock angle” (straight–weak). However, whether this score described the front limbs only, or a combination of front and hind limbs, is not specified. Both measurements of the fetlock joint of the front limb had a higher h^2^ than the scored “fetlock angle” (h^2^ = 0.11, [22]), while the fetlock joint of the hind limb was less heritable when measured within the joint, and more heritable when measured in front of the joint (Table 5). At minimum, selection on shoulder and fetlock angles might therefore be improved by applying the horse shape space model. 

Furthermore, it was demonstrated that horse shape space data can be successfully applied to assess the evolution of a breed over time. The FM stallion population evolved from a heavy to a light draught horse type. The absence of a plateau in more recent years is a cause for concern, as the breed may lose its breed-specific type of a light draught horse. Some changes in the trajectories of the trend lines seemed to coincide with recent introgressions from lighter breeds. More specifically, the changes in the poll angle starting in the 1980s may be a consequence of the Swedish Warmblood introgression in the 1970s, while changes in most of the measurements from the 1990s onward in most of the measurements may be due to the two Swiss Warmblood stallions that were introgressed in 1990. The current favoured use of stallions with high admixture proportions from the last introgressions in FM breeding may accelerate the observed changes in conformation.

Apart from the small sample size, there were some additional limitations to our study. Some stallions from the 1940s and 1950s were only distantly related to the more recent population, which might affect estimates of the variance components due to gaps in the pedigree. Furthermore, all the horses born before 2004 were stallions, due to the low availability of mare photographs in the archives of the Swiss National Stud Farm. However, this allowed us to increase our sample size and to retrace the evolution of the FM conformation traits over time. Whether the FM breeding association will implement the horse shape space model in their selection programme depends on several points: social acceptance by the breeders, practical considerations (time to take the appropriate photographs) and technical feasibility (automation of landmark placement). One further limitation to this study is that we could not provide in-depth analysis of the type of conformation favourable to specific disciplines, as was investigated in other morphometric studies [35,36]. This could be the subject of a future study. 

## 5. Conclusions

Joint angles such as the shoulder and fetlock angles had higher heritability based on the horse shape space model than when based on the scores from linear profiling. In the front limbs, landmark placement within the centre of the joints yielded more reliable and heritable results, while the results were less consistent for the hind limb joint angles. The FM horse breed has evolved from a heavy draught breed to a much lighter type. Care must be taken to not lighten the breed beyond the breeding goal of a light draught horse.

## Figures and Tables

**Table 1:** Landmark placement determining the pairs of joint angles calculated from the horse shape space data.

Trait	Landmark Placement in Front of the Limb [26]	Landmark Placement within the Limb
**Elbow joint angle**	Greater tubercle of the humerus (point of shoulder)—lateral epicondyle of the humerus—anterior aspect of the metacarpal tuberosity of the 3rd metacarpal bone	Greater tubercle of the humerus (point of shoulder)—lateral epicondyle of the humerus—lateral aspect of the carpal ulnar bone
**Carpal joint angle**	Lateral epicondyle of the humerus—anterior aspect of the proximal tuberosity of the 3rd metacarpal bone—anterior aspect of the fetlock joint	Lateral epicondyle of the humerus—lateral aspect of the carpal ulnar bone—lateral aspect of the fetlock joint
**Fetlock joint angle of the front limb**	Anterior aspect of the proximal tuberosity of the 3rd metacarpal bone—anterior aspect of the fetlock joint—anterior aspect of the coronet	Lateral aspect of the carpal ulnar bone—lateral aspect of the fetlock—lateral aspect of the coronet
**Hip joint angle**	Sacral tuber of the ilium (highest point of the croup)—tuber of the ischium (point of buttock)—apex of the patella	Sacral tuber of the ilium (highest point of the croup)—tuber of the ischium (point of buttock)—lateral condyle of the tibia
**Stifle joint angle**	Tuber of the ischium (point of buttock)—apex of the patella—anterior aspect of the tarsus	Tuber of the ischium (point of buttock)—lateral condyle of the tibia—4th tarsal bone
**Hock joint angle**	Apex of the patella—anterior aspect of the tarsus—anterior aspect of the fetlock joint	Lateral condyle of the tibia—4th tarsal bone—lateral aspect of the fetlock joint
**Fetlock joint angle of the hind limb**	Anterior aspect of the tarsus—anterior aspect of the fetlock joint—anterior aspect of the coronet	4th tarsal bone—lateral aspect of the fetlock joint—lateral aspect of the coronet

**Table 2:** Descriptive statistics and repeatability for photographs analysed in triplicate for each trait as an intra-class correlation coefficient with their 95% confidence intervals, for the joint angles.

Trait	Mean	SD	Min	Max	Range	ICC (N = 480)	Low CI	High CI
**Poll**	103.35	5.27	85.90	120.98	35.08	0.99	0.98	0.99
**Neck–shoulder blade**	83.12	5.47	66.72	101.51	34.79	0.95	0.94	0.95
**Shoulder joint**	103.87	4.43	90.71	118.24	27.53	0.81	0.79	0.84
**Elbow joint**	137.90	4.42	120.70	151.40	30.70	0.86	0.84	0.88
**Elbow joint (in)**	143.00	4.53	126.20	156.40	30.20	0.86	0.83	0.88
**Carpal joint**	180.50	1.98	173.10	187.50	14.40	0.68	0.64	0.72
**Carpal joint (in)**	181.30	2.06	172.00	187.50	15.50	0.67	0.63	0.71
**Fetlock joint of the front limb**	148.90	4.07	136.80	161.90	25.10	0.66	0.62	0.70
**Fetlock joint of the front limb (in)**	151.10	4.27	138.40	165.20	26.80	0.74	0.71	0.77
**Hip joint**	78.72	3.10	70.58	91.04	20.46	0.90	0.88	0.91
**Hip joint (in)**	95.88	3.49	106.24	117.19	10.95	0.92	0.91	0.93
**Stifle joint**	100.65	4.08	86.56	113.24	26.68	0.89	0.87	0.90
**Stifle joint (in)**	135.50	3.86	123.00	148.50	25.50	0.89	0.87	0.90
**Hock joint**	153.30	2.32	144.10	160.50	16.40	0.83	0.80	0.85
**Hock joint (in)**	161.80	2.78	152.00	171.70	19.70	0.78	0.75	0.81
**Fetlock joint of the hind limb**	157.20	5.07	135.30	177.80	42.50	0.77	0.74	0.80
**Fetlock joint of the hind limb (in)**	160.60	5.00	138.90	182.20	43.30	0.82	0.79	0.84

**Table 3:** Summary table of the significance of the postural variables, sex, age and year of birth on the different joint angles based on linear regression.

Trait	Head height	Head in relation to camera	Position of front limb	Position of hind limb	Body alignment	Sex (gelding)	Sex (stallion)	Age	Year of Birth
**Poll**		***							***
**Neck–shoulder blade**	***		***		***			***	***
**Shoulder joint**			***				***	*	***
**Elbow joint**	***		***		*		***	**	***
**Elbow joint (in)**	***		***		*		***	**	***
**Carpal joint**					***		***	**	***
**Carpal joint (in)**					***	*	***	**	
**Fetlock joint of the front limb**				**			**		***
**Fetlock joint of the front limb (in)**				**				*	
**Hip joint**				***	***		***		***
**Hip joint (in)**				***	***		***		***
**Stifle joint**			***	***	***	*	***	**	*
**Stifle joint (in)**		**	**	***	***		**	*	
**Hock joint**			*	***	*				
**Hock joint (in)**			**	***	**	**	***		*
**Fetlock joint of the hind limb**			**						**
**Fetlock joint of the hind limb (in)**		*	**		*				

* = *p*-value < 0.05, ** = *p*-value < 0.01, *** = *p*-value < 0.001.

**Table 4:** Summary table of the significance of the postural variables, sex, age and year of birth on the different joint angles based on linear regression.

Trait	Head Height	Head in Relation to Camera	Position of Front Limb	Position of Hind Limb	Body Alignment	Sex (Gelding)	Sex (Stallion)	Age	Year of Birth
**PC1**	***	*	*	*	*		***	**	**
**PC2**	**	***		*	***		***	***	
**PC3**	***		***	***	***			*	***
**PC4**	***		***	***	***		***		
**PC5**			*	***	***		***		

* = *p*-value < 0.05, ** = *p*-value < 0.01, *** = *p*-value < 0.001.

**Table 5:** Heritability with corresponding standard errors, additive genetic (σ_a_), phenotypic (σ_p_) and residual (σ_e_) variances.

Trait	h^2^	SE	σ_a_	σ_p_	σ_e_
**Poll**	0.37	0.09	8.75	23.96	15.21
**Neck–shoulder blade**	0.20	0.08	4.26	21.62	17.36
**Shoulder joint**	0.18	0.06	2.85	15.64	12.79
**Elbow joint**	0.20	0.07	3.55	17.93	14.38
**Elbow joint (in)**	0.19	0.07	3.67	18.95	15.28
**Carpal joint**	0.13	0.07	0.17	1.39	1.21
**Carpal joint (in)**	0.27	0.09	0.52	1.88	1.36
**Fetlock joint of the front limb**	0.29	0.08	4.35	15.71	11.18
**Fetlock joint of the front limb (in)**	0.31	0.08	4.65	18.08	12.43
**Hip joint**	0.23	0.07	1.82	7.95	6.13
**Hip joint (in)**	0.17	0.07	1.50	8.66	7.16
**Stifle joint**	0.08	0.05	0.98	11.79	10.81
**Stifle joint (in)**	0.12	0.06	1.49	12.15	10.66
**Hock joint**	0.16	0.07	0.77	4.86	4.09
**Hock joint (in)**	0.06	0.04	0.41	6.58	6.17
**Fetlock joint of the hind limb**	0.19	0.07	4.17	21.99	17.82
**Fetlock joint of the hind limb (in)**	0.09	0.05	2.05	21.98	19.93

**Table 6:** Genetic correlations (above the diagonal), heritabilities (in the grey-coloured diagonal) and phenotypic correlations with their standard errors in subscript below the diagonal, for joint angle data derived from the horse shape space model.

	Poll	Neck	Shoulder	Elbow	Elbow (in)	Carpal joint	Carpal Joint (in)	Fetlock Front	Fetlock Front (in)	Hip	Hip (in)	Stifle	Stifle (in)	Hock	Hock (in)	Fetlock Hind	Fetlock Hind (in)
**Poll**	0.37_0.09_	−0.34_0.18_	0.02_0.15_	0.28_0.18_	0.26_0.18_	−0.12_0.19_	−0.08_0.17_	0.10_0.18_	0.21_0.17_	−0.36_0.18_	−0.24_0.20_	−0.28_0.22_	−0.04_0.21_	0.05_0.22_	0.13_0.25_	0.14_0.19_	0.14_0.22_
**Neck**	−0.23_0.04_	0.20_0.08_	−0.14_0.21_	−0.62_0.21_	−0.58_0.21_	0.31_0.27_	0.06_0.23_	−0.33_0.23_	−0.42_0.21_	0.18_0.25_	−0.11_0.27_	0.10_0.34_	−0.43_0.26_	0.25_0.27_	−0.03_0.36_	−0.01_0.25_	−0.11_0.30_
**Shoulder**	−0.07_0.04_	0.25_0.04_	0.18_0.06_	0.40_0.19_	0.43_0.18_	−0.37_0.18_	−0.37_0.18_	0.20_0.23_	0.37_0.20_	0.30_0.23_	0.32_0.23_	0.40_0.23_	0.37_0.22_	0.25_0.25_	0.28_0.24_	−0.18_0.22_	0.11_0.25_
**Elbow**	−0.11_0.04_	−0.05_0.04_	0.63_0.03_	0.20_0.07_	0.99_0.00_	−0.60_0.25_	−0.57_0.19_	0.18_0.24_	0.57_0.19_	0.13_0.26_	0.30_0.27_	0.36_0.36_	0.60_0.27_	0.25_0.29_	0.28_0.38_	−0.44_0.24_	−0.04_0.32_
**Elbow (in)**	−0.11_0.04_	−0.05_0.04_	0.64_0.03_	0.99_0.00_	0.19_0.07_	−0.63_0.24_	−0.61_0.19_	0.22_0.24_	0.62_0.19_	0.18_0.26_	0.32_0.27_	0.41_0.35_	0.60_0.27_	0.32_0.29_	0.35_0.36_	−0.41_0.24_	0.02_0.32_
**Carpal joint**	−0.02_0.04_	0.00_0.04_	−0.04_0.04_	−0.14_0.04_	−0.13_0.04_	0.13_0.07_	0.51_0.22_	−0.51_0.26_	−0.71_0.22_	−0.34_0.31_	−0.43_0.32_	−0.49_0.34_	−0.56_0.31_	−0.34_0.31_	−0.53_0.28_	0.02_0.29_	−0.40_0.29_
**Carpal joint (in)**	0.02_0.04_	0.02_0.04_	−0.11_0.04_	−0.22_0.04_	−0.25_0.04_	0.45_0.03_	0.27_0.08_	−0.11_0.22_	−0.49_0.17_	−0.24_0.20_	−0.16_0.25_	−0.45_0.30_	−0.24_0.28_	−0.71_0.19_	−0.57_0.25_	0.25_0.23_	−0.17_0.28_
**Fetlock front**	0.00_0.04_	−0.06_0.04_	0.02_0.04_	0.03_0.04_	0.03_0.04_	−0.15_0.04_	−0.07_0.04_	0.29_0.08_	0.83_0.08_	0.30_0.22_	0.51_0.22_	0.30_0.32_	0.61_0.22_	−0.09_0.27_	0.53_0.33_	0.62_0.17_	0.87_0.15_
**Fetlock front (in)**	0.05_0.04_	−0.10_0.04_	−0.01_0.04_	0.01_0.04_	0.02_0.04_	−0.02_0.04_	−0.16_0.04_	0.69_0.02_	0.31_0.08_	0.26_0.21_	0.42_0.23_	0.40_0.30_	0.61_0.22_	0.32_0.25_	0.73_0.23_	0.33_0.21_	0.76_0.18_
**Hip**	−0.11_0.04_	0.08_0.04_	0.12_0.04_	0.07_0.04_	0.07_0.04_	−0.04_0.04_	−0.02_0.04_	−0.02_0.04_	−0.03_0.04_	0.23_0.07_	0.93_0.05_	0.96_0.09_	0.73_0.19_	0.01_0.29_	0.11_0.39_	−0.35_0.24_	0.02_0.32_
**Hip (in)**	−0.10_0.04_	0.08_0.04_	0.09_0.04_	0.01_0.04_	0.00_0.04_	−0.06_0.04_	−0.01_0.04_	−0.03_0.04_	−0.02_0.04_	0.84_0.01_	0.17_0.07_	0.88_0.18_	0.92_0.11_	−0.21_0.30_	0.08_0.41_	−0.25_0.27_	0.11_0.33_
**Stifle**	0.00_0.04_	0.05_0.04_	0.05_0.04_	0.00_0.04_	−0.01_0.04_	−0.01_0.04_	0.06_0.04_	0.06_0.04_	0.05_0.04_	0.52_0.03_	0.24_0.04_	0.08_0.05_	0.75_0.17_	0.24_0.38_	0.28_0.42_	−0.42_0.33_	0.05_0.40_
**Stifle (in)**	0.03_0.04_	0.02_0.04_	0.02_0.04_	−0.05_0.04_	−0.06_0.04_	−0.03_0.04_	0.09_0.04_	0.09_0.04_	0.09_0.04_	0.39_0.04_	0.41_0.03_	0.80_0.02_	0.12_0.06_	−0.22_0.34_	0.15_0.40_	−0.22_0.29_	0.17_0.35_
**Hock**	0.04_0.04_	−0.02_0.04_	0.05_0.04_	0.01_0.04_	0.00_0.04_	0.00_0.04_	0.07_0.04_	0.05_0.04_	0.13_0.04_	0.15_0.04_	0.11_0.04_	0.36_0.04_	0.37_0.04_	0.16_0.07_	0.68_0.23_	−0.08_0.29_	0.24_0.35_
**Hock (in)**	0.08_0.04_	−0.08_0.04_	0.03_0.04_	0.05_0.04_	0.04_0.04_	−0.01_0.04_	0.06_0.04_	0.09_0.04_	0.11_0.04_	0.10_0.04_	0.08_0.04_	0.34_0.04_	0.54_0.04_	0.74_0.03_	0.06_0.04_	0.37_0.37_	0.72_0.30_
**Fetlock hind**	0.05_0.04_	0.08_0.04_	−0.06_0.04_	−0.06_0.04_	−0.06_0.04_	−0.03_0.04_	0.00_0.04_	0.19_0.04_	0.12_0.04_	0.04_0.04_	0.02_0.04_	0.03_0.04_	0.00_0.04_	−0.09_0.04_	−0.07_0.04_	0.19_0.07_	0.83_0.11_
**Fetlock hind (in)**	0.06_0.04_	0.07_0.04_	−0.02_0.04_	−0.08_0.04_	−0.08_0.04_	−0.05_0.04_	−0.01_0.04_	0.15_0.04_	0.20_0.04_	0.12_0.04_	0.09_0.04_	0.11_0.04_	0.06_0.04_	0.05_0.04_	−0.14_0.04_	0.78_0.02_	0.09_0.05_

**Table 7:** Heritabilities with corresponding standard errors, additive genetic (σ_a_), phenotypic (σ_p_) and residual (σ_e_) variances of the first five relative warp scores (PC1–PC5).

Trait	h^2^	SE	σ_a_	σ_p_	σ_e_
**PC1**	0.13	0.08	4.59 × 10^−5^	3.64 × 10^−4^	3.18 × 10^−4^
**PC2**	0.14	0.08	3.80 × 10^−5^	2.71 × 10^−4^	2.33 × 10^−4^
**PC3**	0.36	0.09	2.89 × 10^−5^	7.97 × 10^−5^	5.08 × 10^−5^
**PC4**	0.29	0.10	3.04 × 10^−5^	1.04 × 10^−4^	7.31 × 10^−5^
**PC5**	0.37	0.09	2.58 × 10^−5^	6.93 × 10^−5^	4.35 × 10^−5^

**Table 8:** Genetic correlations (above the diagonal), heritabilities (in the grey-coloured diagonal) and phenotypic correlations with their standard errors (below the diagonal) for the relative warp scores derived from the horse shape space model.

	PC1	PC2	PC3	PC4	PC5
**PC1**	**0.13_0.08_**	−0.55_0.43_	0.27_0.28_	−0.36_0.39_	0.34_0.30_
**PC2**	−0.09_0.04_	**0.14_0.08_**	0.31_0.29_	0.28_0.34_	−0.51_0.28_
**PC3**	0.41_0.04_	0.06_0.04_	**0.36_0.09_**	−0.24_0.34_	−0.14_0.21_
**PC4**	0.12_0.04_	0.09_0.04_	−0.09_0.04_	**0.29_0.10_**	−0.06_0.23_
**PC5**	0.08_0.04_	−0.07_0.04_	−0.06_0.04_	−0.13_0.04_	**0.37_0.09_**

## Data Availability

As some of the photographed horses belong to private owners, data are only available on reasonable request to the authors.

## Supplementary Materials

The following supporting information can be downloaded at: https://www.mdpi.com/article/10.3390/ani12172186/s1, File S1: Summary of fixed effects estimates for joint angles, relative warp components, postural variables, age, sex and year of birth; Figure S1: Horse shape space model with 246 landmarks on the photograph (a) and without background (b); Figure S2: Evolution of joint angle measurements of the poll angle (a), neck–shoulder blade angle (b) and shoulder joint angle (c) in Franches-Montagnes stallions born between 1940 and 2018; Figure S3: Evolution of joint angle measurements of the front limbs in Franches-Montagnes stallions born between 1940 and 2018; Figure S4: Evolution of joint angle measurements of the hindquarters in Franches-Montagnes stallions born between 1940 and 2018; Figure S5: Evolution of the first five relative warp scores in Franches-Montagnes stallions born between 1940 and 2018.
