# Supplementary material for: Estimates of Genetic Parameters for Shape Space Data in Franches-Montagnes Horses
Source: Animals (Basel). 2022 Aug 25;12(17):2186. doi: 10.3390/ani12172186 (PMC9454882; doi:10.3390/ani12172186)
Supplement: Supplementary file 1 [file animals-12-02186-s001.zip › PDF/Figure S4.pdf]

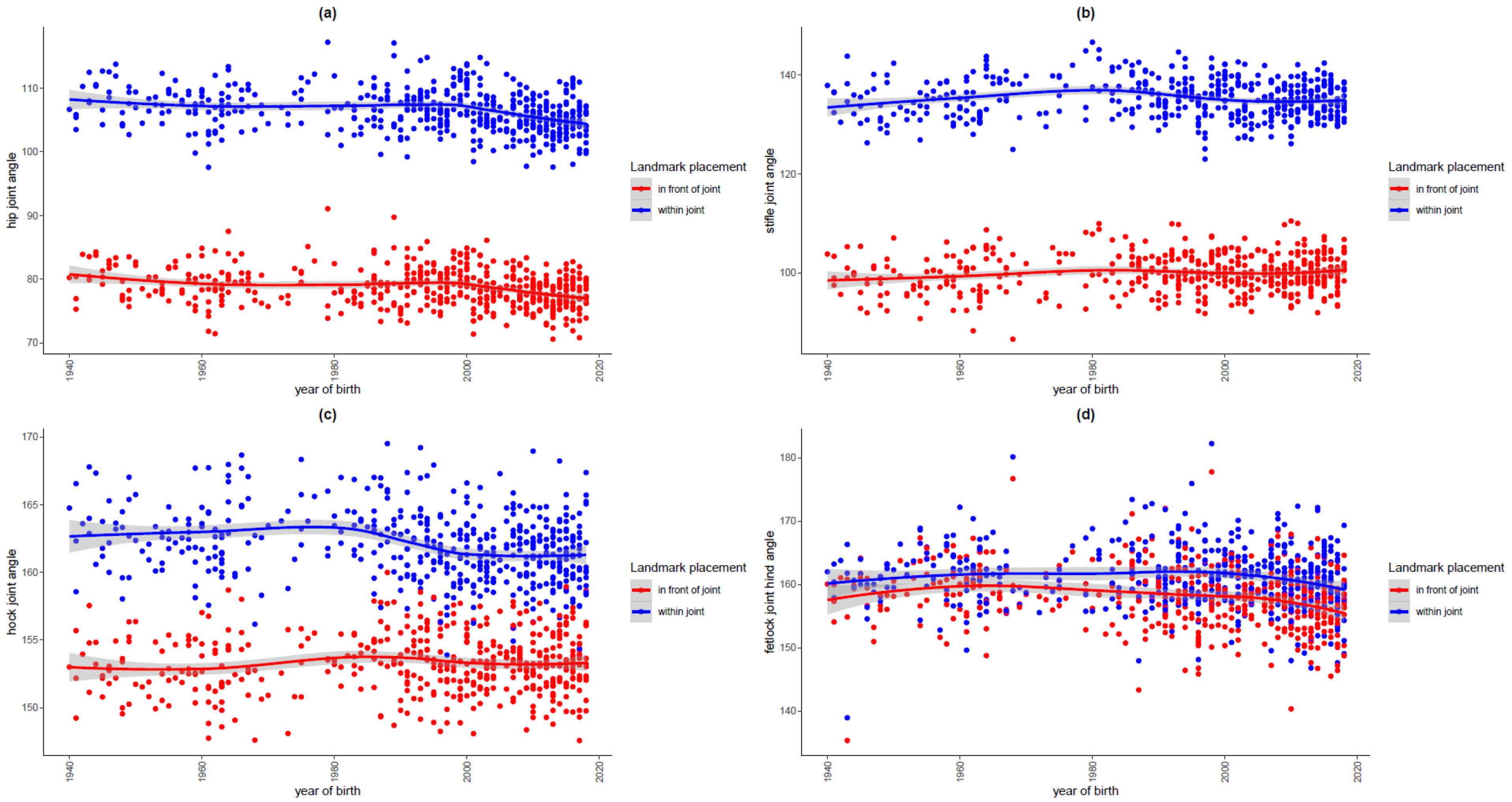

Figure S4: Evolution of joint angle measurements of the hindquarters in Franches-Montagnes stallions born between 1940 and 2018: a) hip joint, b) stifle joint, c) hock joint, d) fetlock joint of the hind limb, with the trend line (in blue, with the confidence interval in light grey) from local polynomial regression fitting.
