# Supplementary material for: Estimates of Genetic Parameters for Shape Space Data in Franches-Montagnes Horses
Source: Animals (Basel). 2022 Aug 25;12(17):2186. doi: 10.3390/ani12172186 (PMC9454882; doi:10.3390/ani12172186)
Supplement: Supplementary file 1 [file animals-12-02186-s001.zip › PDF/Figure S5.pdf]

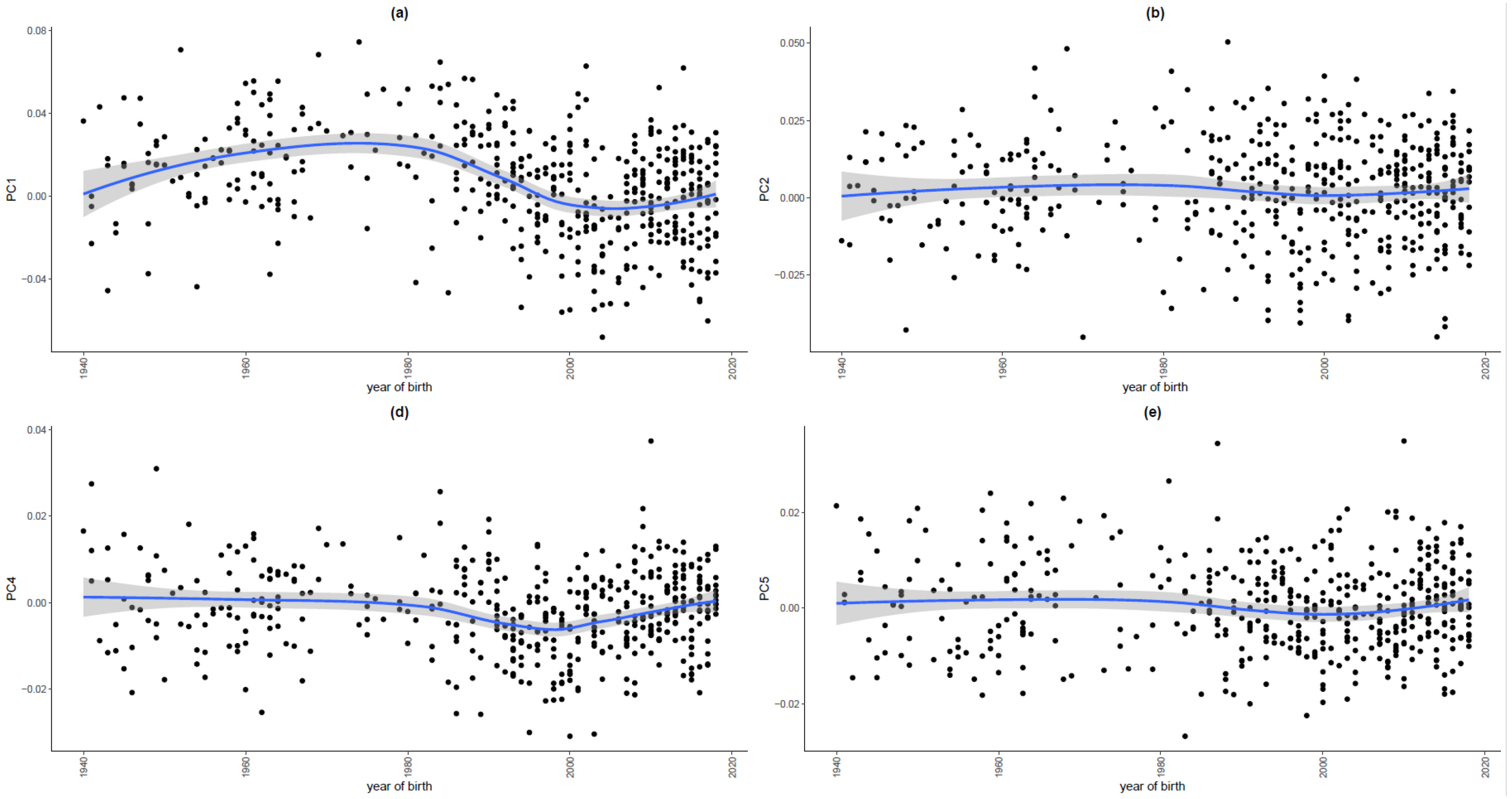

Figure S5: Evolution of relative warp scores in Franches-Montagnes stallions born between 1940 and 2018: a) PC1, b) PC2, c) PC4, d) PC5, with the trend line (in blue, with the confidence interval in light grey) from local polynomial regression fitting.
