# Supplementary material for: Estimates of Genetic Parameters for Shape Space Data in Franches-Montagnes Horses
Source: Animals (Basel). 2022 Aug 25;12(17):2186. doi: 10.3390/ani12172186 (PMC9454882; doi:10.3390/ani12172186)
Supplement: Supplementary file 1 [file animals-12-02186-s001.zip › PDF/File S1.pdf]

File S1

S1.1 Poll angle

Summary of fixed effects estimates

	Estimate	Std. Error	t value	Pr(>|t|)
(Intercept)	241.1716	30.63599	7.872	2.47E-14
Age	0.18216	0.09813	1.856	0.0641
Head_height	-0.37911	0.65022	-0.583	0.5601
Head_camera	1.28345	0.27672	4.638	4.58E-06
Front_limb	0.20937	0.25455	0.823	0.4112
Hind_limb	0.30274	0.28573	1.06	0.2899
Body	-0.42556	0.4604	-0.924	0.3558
YOB	-0.07067	0.01527	-4.628	4.79E-06
Sexg	-1.02843	0.86335	-1.191	0.2342
Sexs	-0.38423	0.7362	-0.522	0.602

S1.2 Neck-shoulderblade angle

Summary of fixed effects estimates

	Estimate	Std. Error	t value	Pr(>|t|)
(Intercept)	-104.403	29.21216	-3.574	0.000388
Age	-0.34653	0.09357	-3.703	2.38E-04
Head_height	-6.05358	0.62	-9.764	< 2e-16
Head_camera	0.06539	0.26386	0.248	0.804371
Front_limb	-0.86244	0.24271	-3.553	4.19E-04
Hind_limb	0.51545	0.27245	1.892	0.059123
Body	1.92678	0.439	4.389	1.41E-05
YOB	0.09721	0.01456	6.677	6.97E-11
Sexg	0.37447	0.82322	0.455	6.49E-01
Sexs	0.87578	0.70199	1.248	0.212814

S1.3 Shoulder joint angle

Summary of fixed effects estimates

	Estimate	Std. Error	t value	Pr(>|t|)
(Intercept)	-24.4183	24.25151	-1.007	0.314516
Age	-0.18607	0.07768	-2.395	1.70E-02
Head_height	-0.27377	0.51471	-0.532	0.595053
Head_camera	-0.23079	0.21905	-1.054	0.292618
Front_limb	0.6879	0.2015	3.414	6.96E-04
Hind_limb	0.17918	0.22618	0.792	0.428659
Body	0.35724	0.36445	0.98	3.27E-01
YOB	0.06454	0.01209	5.34	1.46E-07
Sexg	-0.91933	0.68343	-1.345	1.79E-01
Sexs	-2.5334	0.58278	-4.347	1.70E-05

S1.4 Elbow joint angle

Summary of fixed effects estimates

	Estimate	Std. Error	t value	Pr(>|t|)
(Intercept)	265.5907	25.14332	10.563	< 2e-16
Age	-0.26062	0.08054	-3.236	1.30E-03
Head_height	-2.59472	0.53364	-4.862	1.59E-06
Head_camera	-0.01383	0.22711	-0.061	0.9515
Front_limb	-1.28772	0.20891	-6.164	1.54E-09
Hind_limb	-0.29809	0.2345	-1.271	0.2043
Body	-0.8344	0.37786	-2.208	2.77E-02
YOB	-0.0559	0.01253	-4.461	1.03E-05
Sexg	-1.02187	0.70856	-1.442	1.50E-01
Sexs	-4.15617	0.60421	-6.879	1.96E-11

S1.5 Elbow joint angle (landmarks inside joints)

Summary of fixed effects estimates

	Estimate	Std. Error	t value	Pr(>|t|)
(Intercept)	277.138	25.82302	10.732	< 2e-16
Age	-0.25732	0.08272	-3.111	1.98E-03
Head_height	-2.59673	0.54807	-4.738	2.87E-06
Head_camera	-0.02301	0.23324	-0.099	0.92146
Front_limb	-1.29993	0.21456	-6.059	2.84E-09
Hind_limb	-0.29462	0.24084	-1.223	0.22183
Body	-0.88661	0.38807	-2.285	2.28E-02
YOB	-0.05889	0.01287	-4.576	6.10E-06
Sexg	-1.05947	0.72771	-1.456	1.46E-01
Sexs	-4.52817	0.62054	-7.297	1.28E-12

S1.6 Carpal joint angle

Summary of fixed effects estimates

	Estimate	Std. Error	t value	Pr(>|t|)
(Intercept)	233.4741	11.58862	20.147	< 2e-16
Age	-0.11667	0.037121	-3.143	1.78E-03
Head_height	0.301424	0.245957	1.226	2.21E-01
Head_camera	-0.17158	0.104673	-1.639	0.101859
Front_limb	0.013811	0.096286	0.143	8.86E-01
Hind_limb	-0.12048	0.108081	-1.115	0.265539
Body	-0.57808	0.174155	-3.319	9.73E-04
YOB	-0.0245	0.005776	-4.241	2.68E-05
Sexg	-0.59228	0.326577	-1.814	7.04E-02
Sexs	-1.87859	0.278482	-6.746	4.53E-11

S1.7 Carpal joint angle (landmarks inside joints)

Summary of fixed effects estimates

	Estimate	Std. Error	t value	Pr(>|t|)
(Intercept)	203.6879	12.27178	16.598	< 2e-16
Age	-0.11065	0.039309	-2.815	5.09E-03
Head_height	0.131138	0.260456	0.503	6.15E-01
Head_camera	-0.09869	0.110844	-0.89	0.373736
Front_limb	-0.03413	0.101962	-0.335	7.38E-01
Hind_limb	-0.07344	0.114452	-0.642	0.521435
Body	-0.69805	0.184422	-3.785	1.74E-04
Sexg	-0.87398	0.345829	-2.527	1.18E-02
Sexs	-2.00539	0.294899	-6.8	3.22E-11
YOB	-0.00891	0.006116	-1.456	1.46E-01

S1.8 Fetlock joint of the forelimb

Summary of fixed effects estimates

	Estimate	Std. Error	t value	Pr(>|t|)
(Intercept)	241.1793	24.84248	9.708	< 2e-16
Age	0.100857	0.079576	1.267	2.06E-01
Head_height	-0.00121	0.527257	-0.002	9.98E-01
Head_camera	-0.05605	0.224388	-0.25	0.802857
Front_limb	0.28871	0.206408	1.399	1.63E-01
Hind_limb	0.640551	0.231693	2.765	0.005925
Body	-0.52407	0.373336	-1.404	1.61E-01
YOB	-0.04586	0.012381	-3.704	2.38E-04
Sexg	-0.9126	0.700082	-1.304	1.93E-01
Sexs	-1.83367	0.59698	-3.072	2.25E-03

S1.9 Fetlock joint of the forelimb (landmarks inside joints)

Summary of fixed effects estimates

	Estimate	Std. Error	t value	Pr(>|t|)
(Intercept)	196.4598	26.84227	7.319	1.11E-12
Age	0.211335	0.085982	2.458	1.43E-02
Head_height	0.406778	0.5697	0.714	4.76E-01
Head_camera	0.009479	0.242451	0.039	0.96883
Front_limb	-0.04512	0.223024	-0.202	8.40E-01
Hind_limb	0.670365	0.250344	2.678	0.00767
Body	-0.30454	0.403389	-0.755	4.51E-01
YOB	-0.02345	0.013378	-1.753	8.03E-02
Sexg	-0.81302	0.756437	-1.075	2.83E-01
Sexs	-1.05442	0.645037	-1.635	1.03E-01

S1.10 Hip joint angle

Summary of fixed effects estimates

	Estimate	Std. Error	t value	Pr(>|t|)
(Intercept)	154.7431	16.88321	9.166	< 2e-16
Age	-0.01757	0.054081	-0.325	7.46E-01
Head_height	0.489853	0.358329	1.367	1.72E-01
Head_camera	0.089267	0.152496	0.585	0.5586
Front_limb	0.128539	0.140277	0.916	3.60E-01
Hind_limb	-1.44735	0.157461	-9.192	< 2e-16
Body	1.343888	0.253723	5.297	1.82E-07
YOB	-0.0381	0.008415	-4.528	7.57E-06
Sexg	-0.84816	0.475783	-1.783	7.53E-02
Sexs	-2.55807	0.405714	-6.305	6.70E-10

S1.11 Hip joint angle (landmarks within joints)

Summary of fixed effects estimates

	Estimate	Std. Error	t value	Pr(>|t|)
(Intercept)	193.2437	18.41669	10.493	< 2e-16
Age	-0.09364	0.058993	-1.587	1.13E-01
Head_height	0.076766	0.390876	0.196	8.44E-01
Head_camera	0.258056	0.166348	1.551	0.122
Front_limb	-0.04755	0.153019	-0.311	7.56E-01
Hind_limb	-1.8865	0.171763	-10.983	< 2e-16
Body	1.65951	0.276768	5.996	4.06E-09
YOB	-0.04338	0.009179	-4.726	3.03E-06
Sexg	-0.43844	0.518998	-0.845	3.99E-01
Sexs	-1.79505	0.442565	-4.056	5.85E-05

S1.12 Stifle joint angle

Summary of fixed effects estimates

	Estimate	Std. Error	t value	Pr(>|t|)
(Intercept)	43.49743	20.35857	2.137	3.32E-02
Age	0.20593	0.06521	3.158	1.69E-03
Head_height	0.25756	0.43209	0.596	5.51E-01
Head_camera	0.3376	0.18389	1.836	0.067007
Front_limb	0.61739	0.16915	3.65	2.92E-04
Hind_limb	1.64405	0.18987	8.659	< 2e-16
Body	1.93929	0.30595	6.339	5.49E-10
YOB	0.02268	0.01015	2.235	2.59E-02
Sexg	-1.32872	0.57372	-2.316	2.10E-02
Sexs	-2.43209	0.48923	-4.971	9.37E-07

S1.13 Stifle joint angle (landmarks within joints)

Summary of fixed effects estimates

	Estimate	Std. Error	t value	Pr(>|t|)
(Intercept)	114.3025	20.92364	5.463	7.65E-08
Age	0.151208	0.067023	2.256	2.45E-02
Head_height	-0.2341	0.444083	-0.527	5.98E-01
Head_camera	0.529435	0.188991	2.801	0.0053
Front_limb	0.46754	0.173848	2.689	7.42E-03
Hind_limb	1.002167	0.195144	5.136	4.15E-07
Body	2.539423	0.314443	8.076	5.80E-15
YOB	0.004615	0.010428	0.443	6.58E-01
Sexg	-1.1327	0.589645	-1.921	5.54E-02
Sexs	-1.62482	0.502808	-3.231	1.32E-03

S1.14 Hock joint angle

Summary of fixed effects estimates

	Estimate	Std. Error	t value	Pr(>|t|)
(Intercept)	132.7695	13.6633	9.717	< 2e-16
Age	0.034637	0.043767	0.791	4.29E-01
Head_height	0.291889	0.28999	1.007	3.15E-01
Head_camera	0.091043	0.123413	0.738	0.4611
Front_limb	0.253039	0.113524	2.229	2.63E-02
Hind_limb	-0.90213	0.12743	-7.079	5.37E-12
Body	0.463002	0.205334	2.255	2.46E-02
YOB	0.009919	0.00681	1.457	1.46E-01
Sexg	-0.4354	0.385043	-1.131	2.59E-01
Sexs	-0.44425	0.328338	-1.353	1.77E-01

S1.15 Hock joint angle (landmarks within joints)

Summary of fixed effects estimates

	Estimate	Std. Error t	value	Pr(>|t|)
(Intercept)	200.7653	15.93362	12.6	< 2e-16
Age	0.045903	0.051039	0.899	3.69E-01
Head_height	0.178609	0.338175	0.528	5.98E-01
Head_camera	0.086489	0.143919	0.601	0.548163
Front_limb	0.415378	0.132388	3.138	1.81E-03
Hind_limb	-1.11215	0.148604	-7.484	3.64E-13
Body	0.742079	0.239452	3.099	2.06E-03
YOB	-0.01981	0.007941	-2.495	1.29E-02
Sexg	-1.16335	0.449023	-2.591	9.88E-03
Sexs	-1.2705	0.382895	-3.318	9.77E-04

S1.16 Fetlock joint of the hind limb

Summary of fixed effects estimates

	Estimate	Std. Error	t value	Pr(>|t|)
(Intercept)	242.1572	28.71987	8.432	4.32E-16
Age	-0.06403	0.092	-0.696	4.87E-01
Head_height	-0.88031	0.60955	-1.444	1.49E-01
Head_camera	-0.40909	0.25941	-1.577	0.11548
Front_limb	-0.74789	0.23862	-3.134	1.83E-03
Hind_limb	-0.41299	0.26785	-1.542	1.24E-01
Body	0.21828	0.43161	0.506	6.13E-01
YOB	-0.04036	0.01431	-2.82	5.01E-03
Sexg	-0.45086	0.80935	-0.557	5.78E-01
Sexs	1.0869	0.69016	1.575	1.16E-01

S1.17 Fetlock joint of the hind limb (landmarks inside joints)

Summary of fixed effects estimates

	Estimate	Std. Error	t value	Pr(>|t|)
(Intercept)	184.4095	28.99549	6.36	4.83E-10
Age	0.07006	0.09288	0.754	4.51E-01
Head_height	-0.49799	0.6154	-0.809	4.19E-01
Head_camera	-0.59697	0.2619	-2.279	0.0231
Front_limb	-0.76751	0.24091	-3.186	1.54E-03
Hind_limb	-0.21095	0.27043	-0.78	4.36E-01
Body	0.96495	0.43575	2.214	2.73E-02
YOB	-0.0116	0.01445	-0.803	4.23E-01
Sexg	0.24532	0.81712	0.3	7.64E-01
Sexs	1.19121	0.69678	1.71	8.80E-02

S1.18 PC1

Summary of fixed effects estimates

	Estimate	Std. Error	t value	Pr(>|t|)
(Intercept)	3.83E-01	1.20E-01	3.189	0.001524
Age	-1.19E-03	3.85E-04	-3.082	0.002176
Head_height	-4.00E-02	2.55E-03	-15.684	< 2e-16
Head_camera	2.64E-03	1.09E-03	2.429	0.015518
Front_limb	-2.30E-03	9.98E-04	-2.299	0.021924
Hind_limb	-2.51E-03	1.12E-03	-2.238	0.025673
Body	4.13E-03	1.81E-03	2.289	0.022544
YOB	-1.62E-04	5.99E-05	-2.704	0.007099
Sexg	1.23E-03	3.39E-03	0.364	0.715803
Sexs	1.07E-02	2.89E-03	3.708	0.000234

S1.19 PC2

Summary of fixed effects estimates

	Estimate	Std. Error	t value	Pr(>|t|)
(Intercept)	-7.40E-02	9.75E-02	-0.759	0.448337
Age	1.09E-03	3.12E-04	3.483	0.000543
Head_height	5.83E-03	2.07E-03	2.819	0.005016
Head_camera	5.02E-03	8.80E-04	5.705	2.07E-08
Front_limb	-1.54E-03	8.10E-04	-1.901	0.057951
Hind_limb	2.12E-03	9.09E-04	2.335	0.019949
Body	7.04E-03	1.47E-03	4.807	2.07E-06
YOB	5.82E-06	4.86E-05	0.12	0.904755
Sexg	1.96E-03	2.75E-03	0.714	0.475868
Sexs	1.26E-02	2.34E-03	5.378	1.20E-07

S1.20 PC3

Summary of fixed effects estimates

	Estimate	Std. Error	t value	Pr(>|t|)
(Intercept)	-7.29E-01	5.43E-02	-13.416	< 2e-16
Age	-4.32E-04	1.74E-04	-2.485	0.0133
Head_height	-5.25E-03	1.15E-03	-4.554	6.72E-06
Head_camera	-2.60E-04	4.91E-04	-0.529	5.97E-01
Front_limb	-3.91E-03	4.51E-04	-8.667	< 2e-16
Hind_limb	3.60E-03	5.06E-04	7.1	4.68E-12
Body	4.31E-03	8.16E-04	5.282	1.96E-07
YOB	3.66E-04	2.71E-05	13.52	< 2e-16
Sexg	2.12E-03	1.53E-03	1.387	0.166
Sexs	-1.39E-03	1.31E-03	-1.066	2.87E-01

S1.21 PC4

Summary of fixed effects estimates

	Estimate	Std. Error	t value	Pr(>|t|)
(Intercept)	-1.72E-02	6.15E-02	-0.28	0.779741
Age	-1.78E-04	1.97E-04	-0.904	0.366304
Head_height	-5.41E-03	1.31E-03	-4.141	4.10E-05
Head_camera	4.98E-04	5.56E-04	0.895	3.71E-01
Front_limb	1.75E-03	5.11E-04	3.427	0.000663
Hind_limb	2.74E-03	5.74E-04	4.777	2.39E-06
Body	-5.14E-03	9.24E-04	-5.56	4.55E-08
YOB	1.88E-05	3.07E-05	0.612	0.540574
Sexg	-2.48E-03	1.73E-03	-1.43	0.153335
Sexs	-7.92E-03	1.48E-03	-5.356	1.34E-07

S1.22 PC5

Summary of fixed effects estimates

<table>
<tr><th></th><th>Estimate</th><th>Std. Error</th><th>t value</th><th>Pr(>|t|)</th></tr>
<tr><td>(Intercept)</td><td>6.56E-02</td><td>4.99E-02</td><td>1.314</td><td>0.189462</td></tr>
<tr><td>Age</td><td>2.10E-04</td><td>1.60E-04</td><td>1.311</td><td>0.190484</td></tr>
<tr><td>Head_height</td><td>4.85E-04</td><td>1.06E-03</td><td>0.458</td><td>6.47E-01</td></tr>
<tr><td>Head_camera</td><td>-7.28E-04</td><td>4.51E-04</td><td>-1.617</td><td>1.07E-01</td></tr>
<tr><td>Front_limb</td><td>1.02E-03</td><td>4.14E-04</td><td>2.462</td><td>0.014165</td></tr>
<tr><td>Hind_limb</td><td>5.92E-03</td><td>4.65E-04</td><td>12.73</td><td>< 2e-16</td></tr>
<tr><td>Body</td><td>2.61E-03</td><td>7.50E-04</td><td>3.487</td><td>5.34E-04</td></tr>
<tr><td>YOB</td><td>-4.69E-05</td><td>2.49E-05</td><td>-1.888</td><td>0.059658</td></tr>
<tr><td>Sexg</td><td>1.01E-03</td><td>1.41E-03</td><td>0.721</td><td>0.471561</td></tr>
<tr><td>Sexs</td><td>5.08E-03</td><td>1.20E-03</td><td>4.235</td><td>2.75E-05</td></tr>
</table>
