# Supplementary figures and images for: Estimates of Genetic Parameters for Shape Space Data in Franches-Montagnes Horses
Source: Animals (Basel). 2022 Aug 25;12(17):2186. doi: 10.3390/ani12172186 (PMC9454882; doi:10.3390/ani12172186)

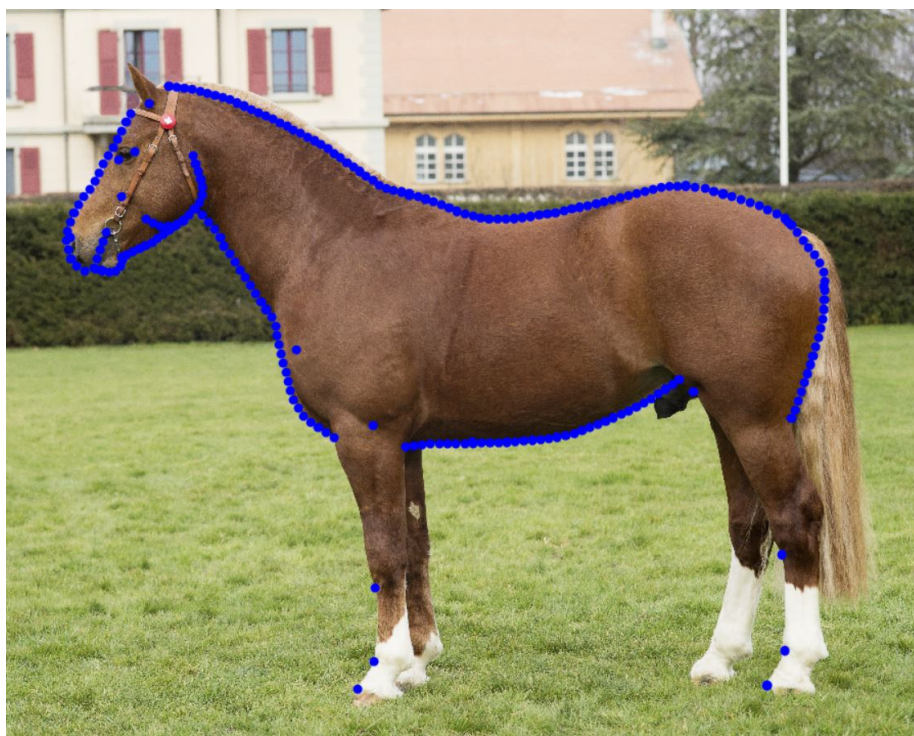

(a)

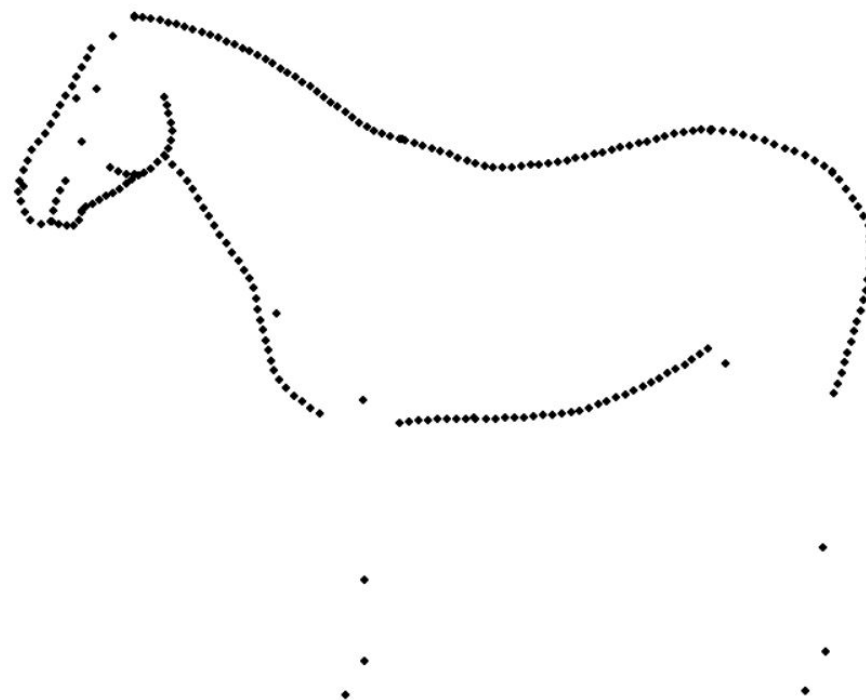

(b)

*Figure S1 : Horse shape space model with 246 landmarks on the photograph (a) and without background (b).*

Supplement: Supplementary file 1 [file animals-12-02186-s001.zip › PDF/Figure S1.pdf]
